# “Once the delivery is done, they have finished”: a qualitative study of perspectives on postnatal care referrals by traditional birth attendants in Ebonyi state, Nigeria

**DOI:** 10.1186/s12884-017-1616-x

**Published:** 2017-12-19

**Authors:** Adanna Chukwuma, Chinyere Mbachu, Jessica Cohen, Thomas Bossert, Margaret McConnell

**Affiliations:** 1 Harvard T.H. Chan School of Public Health, 677 Huntington Avenue, Boston, MA 02115 USA; 2 Health, Nutrition, and Population Global Practice, World Bank Group, Washington, DC 20433 USA; 3 Health Policy Research Group, College of Medicine, University of Nigeria, Enugu, Nigeria

**Keywords:** Postnatal, Traditional birth attendant, Maternal health, Nigeria, Referral

## Abstract

**Background:**

While 79% of Nigerian mothers who deliver in facilities receive postnatal care within 48 h of delivery, this is only true for 16% of mothers who deliver outside facilities. Most maternal deaths can be prevented with access to timely and competent health care. Thus, the World Health Organization, International Confederation of Midwives, and International Federation of Gynecology and Obstetrics recommend that unskilled birth attendants be involved in advocacy for skilled care use among mothers. This study explores postnatal care referral behavior by TBAs in Nigeria, including the perceived factors that may deter or promote referrals to skilled health workers.

**Methods:**

This study collected qualitative data using focus group discussions involving 28 female health workers, TBAs, and TBA delivery clients. The study conceptual framework drew on constructs in Fishbein and Ajzen’s theory of reasoned action onto which we mapped hypothesized determinants of postnatal care referrals described in the empirical literature. We analyzed the transcribed data thematically, and linked themes to the study conceptual framework in the discussion to explain variation in TBA referral behavior across the maternal continuum, from the antenatal to postnatal period.

**Results:**

Differences in TBA referral before, during, and after delivery appear to reflect the TBAs understanding of the added value of skilled care for the client and the TBA, as well as the TBA’s perception of the implications of referral for her credibility as a maternal care provider among her clients. We also found that there are opportunities to engage TBAs in routine postnatal care referrals to facilities in Nigeria by using incentives and promoting a cordial relationship between TBAs and skilled health workers.

**Conclusions:**

Thus, despite the potential negative consequences TBAs may face with postnatal care referrals, there are opportunities to promote these referrals using incentives and promoting a cordial relationship between TBAs and skilled health workers. Further research is needed on the interactions between postnatal maternal complications, TBA referral behavior, and maternal perception of TBA competence.

**Electronic supplementary material:**

The online version of this article (10.1186/s12884-017-1616-x) contains supplementary material, which is available to authorized users.

## Background

Most maternal deaths occur within 48 h of childbirth, predominantly from severe bleeding, hypertensive diseases, and infections [1]. Whether a woman dies in the first 48 h after delivery often depends largely on access to timely and competent care [1]. Therefore, the World Health Organization recommends that health providers retain mothers in health facilities after delivery for at least 24 h, for “regular assessment of vaginal bleeding, uterine contraction, fundal height, temperature, and pulse routinely” beginning at the first hour of birth. In addition, blood pressure measurements are recommended shortly after birth, and six hours after if normal, or more frequently if abnormal. These regular assessments facilitate early diagnosis, testing, treatment, and appropriate referral in the event of delivery complications [2].

In Nigeria, the probability that a mother receives postnatal care within 48 h of delivery varies with place of birth. While 79% of mothers who deliver in facilities receive postnatal care within 48 h of delivery, this is only true for 16% of mothers who deliver outside facilities [3]. On average, a traditional birth attendant (TBA) provides delivery assistance in one in every three non-facility deliveries in Nigeria [3]. A TBA is a non-formally trained and community-based provider of pregnancy-related care that works independently of the health system [4]. The World Health Organization, International Confederation of Midwives, and International Federation of Gynecology and Obstetrics released a joint statement in 2004 recommending that the role for TBAs in formal health care systems be restricted to advocacy for and referrals to skilled maternal health care workers [4].

To facilitate advocacy for skilled care, several programs have trained TBAs to identify clinical features of complications following delivery and refer mothers with these features to health workers. A narrative review and meta-analysis of these programs found 16 studies, of which none randomized the assignment of the training intervention, and 13 had neither control group nor any other counterfactual [5]. Only four studies included either a control group or followed a cohort over time, and the results from these studies suggest that training TBAs may not increase the probability of detecting complications or referral [5]. TBAs in one study reported that they lost credibility as providers of care when a client was referred to skilled providers [6]. Therefore, TBA knowledge of conditions requiring referral may not increase advocacy for skilled care, potentially due to the possibility of losing credibility before clients.

Other factors hypothesized in the literature to encourage TBA postnatal care referral behavior are summarized below (Table 1) including client-specific, TBA-specific, and health worker-specific factors.

**Table 1:** Hypothesized determinants of postnatal care referrals by TBAs

Factor	Condition hypothesized to encourage maternal postnatal care referrals by TBAs
TBA-specific	Ability to recognize obstetric complications [5, 6, 21, 22]; understanding postnatal care is necessary in the absence of obstetric complications [19, 20]; lack of perception of negative social or economic consequences of postnatal referral [5, 6]; monetary rewards for referrals [23, 27, 34]; TBA perception that skilled health worker is better able to manage obstetric complication [13].
Client-specific	Client ability to recognize danger signs of obstetric complications [6]; client perception of TBA competence [5, 6]; positive perception of relative quality of care by skilled providers [21, 22].
Health worker-specific	Health worker training to improve relations with TBAs [21, 22]; other social support from health workers including recognition of TBA’s role [23–25].

In this study, we used focus group discussions (FGD) to explore postnatal care referral behavior by TBAs in Nigeria, by examining perspectives of TBAs, TBA clients, and health workers on the role of TBAs in maternal health care during and after pregnancy as well as the perceived factors that may deter or promote referrals by TBAs to skilled care workers from the pre-pregnancy to postnatal period.

## Methods

### Conceptual framework

The study conceptual framework drew on constructs in Fishbein and Ajzen’s theory of reasoned action [7, 8]. This theory predicts behaviors that are under volitional control, that is where the person exercises control over the behavior. Thus, we applied it to advocacy for skilled care among TBAs. This theory assumes that behavior is predicted by intention, which is determined by attitudes toward and perceptions of social norms regarding the behavior [8]. Attitude is determined by the individual’s beliefs about the results of performing the action, referred to as behavioral beliefs, and the value placed on these results. Thus, if a person believes that the results will be positive, the theory predicts a positive attitude towards that behavior. However, if a person believes that the results will be negative, the theory predicts a negative attitude towards that behavior. Perceptions of social norms are determined by normative beliefs, that is a person’s beliefs about whether significant individuals approve or disapprove of their behavior. If a person believes that these significant individuals think he or she should perform an action, he or she will hold a positive subjective norm. However, if a person believes that these significant individuals think he or she should not perform an action, he or she will hold a negative subjective norm [8].

### Focus group discussions

This study collected data primarily using focus group discussions (FGDs), which are useful for studying phenomena at an aggregate level and for assessing collective opinions [9]. FGDs can also aid in developing interventions to address public health problems that have local meaning and utility. In this case the study team intended to decipher if there was justification for and to inform the design of a field experiment on performance-based monetary incentives for TBA referrals in postnatal care.

### Study context and participants

The study was conducted in July 2016 in Ebonyi State, South-Eastern Nigeria, where about 1 in 2 mothers does not receive postnatal care within the first two days of childbirth [3]. As part of a larger mixed methods study, the study team purposively selected 128 wards in Ebonyi State that had at least one primary health care facility with a health care provider offering maternal postnatal care. We identified these wards using the national facility census list and via consultations with officials in the State Ministry of Health. Each selected ward also had to have at least one TBA who lived and worked there. We recruited FGD participants from these wards. The recommended sample size for focus group discussions is 6–12 participants, as group interviews are difficult to manage above 12 [10]. The study team decided on FGD participant categories based on the key stakeholder groups involved in referrals for postnatal care. Thus, we purposively selected 10 TBAs, 10 TBA clients, and 8 health care providers from communities that had primary health care facilities offering postnatal care services by a skilled provider and at least one resident TBA (we intended to interview 10 health care providers, 2 of whom arrived at the interview venue after the discussion had held). At recruitment, potential participants were informed that the study was aimed at understanding postnatal care practice in their communities. Health care providers were recruited face-to-face through a monthly meeting in the primary health care board in the state capital. TBAs were identified in partnership with health care providers, and recruited face-to-face in the 2 cases where they were based in the state capital. Recruitment over the phone was done for 8 TBAs who lived outside the state capital. We recruited TBA clients by asking recruited TBAs for the name of at least one past client, who was then contacted by the study team. Recruitment over the phone was done for 8 TBA clients who lived outside the state capital. TBA clients were required to be beyond 42 days after the culmination of their first pregnancy so that they would have had the opportunity to choose to attend at least one postnatal visit. TBA clients with multiple past pregnancies were therefore qualified to join the study as well. None of the recruited participants declined the invitation to the study.

### Data collection

We held one FGD with 8 health workers, another with 10 TBAs, and a final FGD with 10 TBA delivery clients (9 of whom came for the discussion with their newborns). Discussions were held in a quiet, secluded location in the State Teaching Hospital. Focus groups were conducted in either English or Igbo language depending on participant consensus. The discussions were audio-recorded with the consent of participants and facilitated using topic guides by an experienced qualitative researcher. One member of the research team took notes describing the group interaction. The topics in the discussion guide were informed by the review of the empirical literature and study conceptual framework and differed by participant type (see Additional file 1).

### Data analysis

The research team translated the audio-recordings in Igbo language into English, and transcribed all the recordings verbatim in Microsoft Word. The transcripts were anonymized with pseudonyms and entered into NVivo V.11. Data analysis began with reading of the transcripts at least twice to achieve immersion. Then, codes were derived by identifying phrases that captured key concepts on which majority of the group agreed on or did not object to, via open coding. That is, we developed codes based on the meaning that emerged from the data. We also identified perspectives that deviated from the group consensus and highlighted them in the analysis. Two team members independently coded the study transcripts. Codes that were similar were grouped into themes. For example, individual factors that influenced client preference for TBAs in the antenatal period were coded separately, and grouped subsequently into one theme [11]. In the discussion, we linked study themes to the study conceptual framework, to explain variation in TBA referral behavior across the maternal continuum, from the antenatal to postnatal period. The two members of the research team involved in facilitating the group discussions and analyzing the data are both medical doctors who have experience providing care at the primary, secondary, and tertiary level, and who have graduate training in public health. To prevent the researchers from imposing opinions informed by their background on the discussants, the study team and experts from other disciplines deliberated on the choice of questions and probes used in the topic guide, as well as the themes from the analysis. These deliberations ensured that the leading questions and probes were avoided in the topic guides and that the discussion was facilitated, analyzed, and interpreted to reflect the opinions of participants rather than the biases of the researchers. The study findings have been reported in line with the consolidated criteria for reporting qualitative research (COREQ) [12].

## Results

All the participants were female. The focus groups lasted an average of 75 min (64–87 min). Most participants (39%) were between 30 and 39 years of age. A slightly lower proportion (36%) were between 20 and 29 years of age.

In our analysis, we identified six themes, discussed below with anonymized quotes in italics. We have ordered these themes to reflect interactions between the TBA, clients, and health workers along the continuum of the maternal care experience, which progresses from the antenatal period, through delivery, to the postnatal period.

### Theme 1: TBAs are the preferred provider for their clients before and during delivery because their care is perceived as relatively competent and cheap

Every client expressed preference for care provided by the TBA over care offered by providers in the primary health center in their community. Responding to a comment by a health worker advocating for restrictions on TBA service provision, another one remarked that most clients in her community preferred care provided by TBAs to services in the facility. This preference for TBAs by several mothers in their communities was acknowledged by the other health workers in the focus group.

The women believe so much in them (TBAs)...Most of them do not even seek for advice from us (health workers), it is only very few of them that do that. (Participant 1, health worker focus group)

The focus group discussions revealed potential explanations for the preference for TBAs among their clients. Two TBA clients noted, and the other clients agreed, that services offered by TBAs were cheaper on average than facility care, and that service affordability was one of the reasons why the TBA was preferred over skilled health workers. One health worker also acknowledged the role that affordability of TBA services may play in maternal care-seeking behavior. In addition, while health facilities required deposits to be made prior to the receipt of care, a woman could pay for the TBA’s services after they were offered. Provision for payment after services were received was mentioned by two clients and acknowledged by the rest of the client group, as well as one TBA. The TBA’s perceived competence on maternal health was another reason for choosing to receive care from the TBA. Every client stated that she trusted her TBA because her baby had been delivered safely. Three clients also stated that they considered the potential for maternal and neonatal complications with TBA delivery care to be low. One client believed the probability of complications to be higher in health facilities than with TBA care, and another client stated that the TBA received referrals from skilled health workers in the event of delivery complications, to buttress the TBA’s competence in service provision. Clients considered the advice given by TBAs to expectant mothers during antenatal care equivalent to that given by skilled health workers. No client in the group objected to the idea that care offered by TBAs was at least as good as facility care.

My mother is a TBA, and even the health workers in the health center call her to assist them when delivery seems difficult for them. She is good and well known in attending to birth, regardless of the part of the body that the baby is coming out with, the baby will be delivered safely. So, everybody works based on their level of knowledge. (Participant 8, client focus group)

Clients also reportedly chose to patronize the TBA because she was considered more respectful than skilled health workers. No client in the group reported receiving disrespectful care from a TBA, but four clients reported negative past experiences in facilities. In one case, a client that had used facility delivery care in the past, chose to use a TBA’s services in a subsequent pregnancy because of the disrespectful care she had received from skilled health providers.

While the TBA’s services were preferred for the above reasons, TBAs believed that the lack of essential medical supplies and equipment constrained their ability to provide better care for their clients. Three TBAs highlighted this point, and one health worker stated that she was helping the TBA in her community to acquire equipment and modern medicines to provide better care.

### Theme 2: TBA clients often receive antenatal and delivery care from both skilled and unskilled heath workers, coordinated by the TBA

All but one TBA reported that before delivery, their clients received care from both the TBA and skilled health workers, usually on the TBA’s advice. During antenatal care, TBAs referred their clients to facilities for vaccinations, ultrasound examinations, and other tests that TBAs could not provide themselves. TBA clients also registered in a maternal health clinic and returned to the TBA with reports from tests and other care received in health facilities. One of the TBAs in the focus group was providing antenatal care unsupervised in the community health center when she was recruited by the research team. Two health workers reported that TBA clients in their communities received care in the health facility in addition to antenatal care provided by the TBA.

When you see a pregnant woman in the community, although you are traditional birth attendant, you'd still ask her whether she has registered for antenatal. Even if she plans delivering at your home, she needs to go for antenatal care and receive the necessary tests and medications. The way I do mine is that I normally insist on seeing the test results to ensure that she is not HIV positive. (Participant 9, TBA focus group)

TBAs functioned as the first level of maternal health care, retaining responsibility for coordinating their clients care throughout pregnancy, and referring cases at their discretion*.* Registering with the local health clinic facilitated referrals of clients to skilled health workers by TBAs during delivery in the event of complications the TBA did not think she could manage. The reasons for which TBAs referred to skilled health workers for delivery care were varied and could be arbitrary, including prolonged labor, a HIV positive status, excessive bleeding, or the suspicion that a labor would be difficult.

If I realize that she is likely going to have bleeding after delivery, I'd advise her against delivering at home but to go to a health facility and deliver there, so that if she eventually bleeds there, they can take care of her there. (Participant 5, TBA focus group)

Two health workers expressed support for the existing arrangement in which the TBA provided care before and during delivery, referring for medication, tests, and complications. However, TBA service provision was criticized by the other health workers, whose remarks centered on the risks that TBA antenatal and delivery practices exposed clients to. One health worker stated that referrals during delivery sometimes occurred when it was too late to intervene*.* Five other health workers also gave examples of TBA practices during the antenatal and delivery care that were potentially harmful including alleged transmission of HIV via unsterilized tools, spreading infection because gloves were not worn, leaving the newborn exposed after delivery with risk of hypothermia, and administering medication contraindicated in pregnancy.

A child delivered by a TBA ... was brought to the health center because he was sick. We found out that the child is HIV positive and the mother is negative...the father was also negative. We had to trace the cause of it, and we found out that the baby was contaminated at the place of delivery. The TBA used the same tools she used for the HIV positive mother to deliver the other woman, and the baby was infected. (Participant 2, health worker focus group)

### Theme 3: The postnatal experience for mothers differs for facility and non-facility deliveries

Every health worker reported that mothers who delivered in their facility were retained for 24–48 h. Two health workers noted that complications could develop unexpectedly informing their decision to retain clients in facilities for regular maternal checks and for neonatal vaccinations. Following discharge, clients were asked to return to the facility six weeks later or earlier if there were signs of complications. The health workers also reviewed the postnatal care they offered at each visit encompassing vital signs, urine voiding, breastfeeding patterns, family planning advice, abdominal pain, and maternal nutrition.

After a woman delivers in my health center and has been monitored for a day or two, and I observe that all is alright with her, and the proper immunizations given to the child, I usually ask her to come back after six weeks... But before she leaves the health center, we would tell her to come back if there is any problem before that six weeks. Postnatal care is important for all women after delivery…because complications after delivery are not something to be predicted. (Participant 1, health worker focus group)

No TBA reported offering postnatal care to her clients following delivery, that is retaining her clients over at least one day and conducting regular assessments to detect complications. No TBA reported referring her clients to skilled health workers for maternal postnatal care. Two health workers stated that TBAs in their communities left immediately after delivering the placenta and baby if the mother and neonate did not develop complications during delivery. There was no objection by other health workers to this claim.

Yes, they (TBAs) end their work when they see that both the mother and the child are alive after delivery most times. Sometimes they are even called to go to the woman’s place and they leave after the delivery of the child. (Participant 3, health worker focus group)

There was a consensus among TBAs about the need for neonatal immunizations after delivery, even though there was variation on the recommended timing of the first immunization visit, from immediately after childbirth to six weeks postnatally. Only one health worker noted that the TBA in her community did not refer mothers for neonatal immunizations, and every TBA client reported visiting the facility postnatally to receive immunizations for her newborn. While the need for immunization was acknowledged by all the TBA clients, only three TBA clients considered postnatal care for the mother to be necessary in addition to immunizations and other care for the newborn. These three clients had received maternal postnatal care, in addition to immunizations for their neonates.

In response to a question about factors that might prevent them from receiving maternal postnatal care in facilities within two days of delivery, the consensus in the client focus group was that cost of transport and treatment, and far distances from the nearest facility were the main barriers to receiving maternal postnatal care. One client noted that these barriers would not prevent postnatal care attendance when a mother developed complications. None of the clients considered cost or distance a barrier to receiving neonatal immunizations in the health facility.

Lack of money could be a reason for not visiting the health facility within two days after delivery. When the money is not there, and the person seem to be strong, they might decide to wait for some time for the money to come, and when the person is not strong, they will look for where to borrow money from before taking you to the health facility. (Participant 8, client focus group)

### Theme 4: Engaging in facility referrals in the postnatal period may negatively affect the TBA

Three TBAs remarked that referring a client who does not have complications during or after delivery would strengthen their reputation as competent at birth attendance, attracting new clientele. There was no objection from other TBAs. On a related note, another TBA remarked that if clients referred following delivery repeatedly had complications, the TBAs reputation would suffer and she would lose clientele.

We all agree that good things are better done well, and we all have said that we are doing the right thing. But when you assist the first a woman in delivery, and the placenta, for instance, did not come out and you hastily take her to the health facility. Then, you assist the second woman and some parts of the baby is not coming out, and after your effort you decided to take her to the health center, and for all these complications, the professionals in the health facility also proffer the solution. Then, in the third person, the woman has severe bleeding, do you think you'd still be attending to birth? (Participant 3, TBA focus group)

In response to questions on the potential influence of a TBA referral to the facility on their perception of the TBA, clients unanimously disagreed with the notion that the TBAs reputation would be negatively influenced if they were referred for delivery complications. TBAs who referred clients for postnatal care, routinely or for complications, were characterized as good, altruistic, and deserving compensation.

We will take her the same way (if she asks us to attend postnatal care in the facility), because she has a reason for asking us to go there, probably the person has a problem that she cannot take care of or that she doesn't have the drugs and the necessary things to treat the person. And whatever they tell us there, we come back to tell her. So, there must have been a problem she noticed before sending us to the place. (Participant 7, client focus group)

### Theme 5: TBAs and health workers perceive that a formal and cordial relationship with health workers would encourage postnatal care referrals

In response to a question on what support from government, the community, health workers, or other stakeholders would enable them refer clients to health facilities for postnatal care, remarks by TBAs highlighted the potential for a cordial and formal working relationship with skilled health workers to increase postnatal care referrals. One TBA suggested that a skilled health worker could be assigned to mentor each TBA, another cited the existing partnership between skilled workers and herself in which she worked in the facility as facilitating referrals, and a third TBA asserted that the fear of being arrested deterred referrals.

I work with the health workers at the health facility. I am not doing it in secret. I am transparent in what I am doing and I send the women to go to the health facility for the ones that I will not be able to do myself. (Participant 10, TBA focus group)

Health workers also recognized that a more cordial relationship with TBAs in their community may increase referrals by TBAs to the facility after delivery. Two health workers cited examples where TBAs were more open to referring their clients after being incorporated into local health decision-making committees or after assisting health workers in facilities. The other health workers had not incorporated TBAs in their communities into the health facility or local decision-making committees. However, two other health workers noted that treating TBAs with condescension was unlikely to encourage facility referrals. There was no objection from other members of the group.

That TBA I told you about (that refers her clients to health workers) is a member of our health committee here, and we usually teach them some of these things during our meeting, and to bring the women to the hospital.... The village committee was selected by the village councilor. (Participant 2, health worker focus group)

### Theme 6: TBAs perceive that incentives would encourage postnatal care referrals

TBAs believed that certain incentives would motivate them to refer clients to health facilities for postnatal care. One TBA highlighted non-financial incentives that could be given as prizes, including recognition for referrals and supplies like diapers and mosquito nets to distribute to their clients; while another TBA stated that equipment for maternal health care would motivate referrals to skilled health workers. Most TBAs agreed with suggestions that might improve the attractiveness of their services to clients such as equipment and gifts for their clients. Unprompted, a health worker also suggested that rewards might motivate TBA referrals.

You should know this more than I do, there are many prizes that can be given. It all depends on what you people can do, because some of these doctors usually write their names under the request paper for ultrasound scan when they send the women to do ultrasound. And I think the essence of that is for the people doing the ultrasound to know that the doctor sent the person and to attend to the person immediately. But when we send them, it is usually different, we should also be recognized there as that will also encourage us. But if you people are financially buoyant enough, you can provide things like babies pampers, or mosquito nets that will help us in our work, you will be happy with that. (Participant 4, TBA focus group)

Several TBAs stated that they would prefer to be paid for referrals. Only one TBA objected and suggested that she would prefer the construction of a home where TBAs could offer their services, a proposal that was rejected by all the other TBAs.

## Discussion

In line with the theory of reasoned action, we find that referral behavior among TBAs reflects her attitudes towards this behavior and her perception of the social implications of engaging referrals. The differences in TBA referral before, during, and after delivery appear to reflect the TBAs understanding of the added value of skilled care for the client and the TBA, as well as the TBA’s perception of the implications of referral for her credibility as a maternal care provider among her clients. In the antenatal period, TBAs in this study routinely asked their clients to register for antenatal care in a local health facility, while receiving nonorthodox antenatal care from the TBA that health workers in the study considered harmful. Registering for antenatal care gave the client access to tests and immunizations, and was also intended to facilitate delivery referrals in the event of complications. In contrast to this finding, other studies in Nigeria and Nepal have reported that TBAs provided unstructured antenatal care [13, 14] and largely did not refer clients to skilled antenatal care providers [15].

TBAs in this study did not think that antenatal referrals had negative implications for their credibility as competent health care providers during the antenatal and delivery period.

This perspective among TBAs about the reputational implications of antenatal care referrals may not be unfounded. Clients in this study considered antenatal and delivery care offered by the TBA to be of high quality despite referrals. Following referrals, clients returned to TBAs as they would to a primary health care worker, such that the TBA was responsible for maternal care coordination. Client confidence in the TBA’s ability to provide quality maternal health services has been reported in other studies in Nigeria and Sierra Leone [16, 17]. Even in studies where a higher proportion of TBA clients reported that TBAs (16%) have poor medical skills relative to skilled providers (0.5%), the majority (61%) of clients were satisfied with TBA delivery care [18]. The positive experiences with TBA care in this study may in part reflect that TBAs directed the study team to past clients that had favorable service impressions.

Referrals to skilled providers postnatally was less systematic than the antenatal period, even in the presence of delivery complications. A similar finding has been reported in Nepal [13]. Unlike antenatal and delivery care, no TBA client in this study received postnatal care from the TBA or was referred to a facility for postnatal checkup in their last pregnancy. Akin to TBAs in our FGDs, qualitative research in Tanzania reveals that TBAs tend to consider postnatal care necessary for neonatal immunizations and for maternal or neonatal complications [19, 20]. This finding highlights that there might still be a role for educating TBAs on the importance of regular postnatal care checkups for all mothers, despite evidence suggesting these training sessions do not increase referral rates [5, 21, 22]. While necessary, educating TBAs may be insufficient to encourage postnatal referrals given potential negative social and economic implications of postnatal care referrals, especially in the event of complications. In Guatemala, TBAs reported that they lost credibility as providers of maternal health care when a client was referred to facilities [6]. In this study, TBAs considered referrals for women who had not developed complications during delivery to be a means of boosting their reputation as competent care providers, attracting new clientele. Conversely, when a mother had complications and had to be referred to skilled health workers who successfully managed these complications, the TBA considered herself at risk of losing credibility. Therefore, TBAs in the study perceived that they may not have incentives to refer a client for postnatal care, even if they were comfortable with referring her for antenatal care. Interestingly, clients in this study directly contradicted this perception by TBAs that postnatal care referrals for complications would make them consider the TBA less competent and less likely to patronize her in future pregnancies. Further research on the interactions between postnatal complications, TBA referral behavior, and maternal perception of TBA competence is needed.

In addition to explaining TBA referral behavior, this study was focused on identifying potential means of increasing referrals among TBAs and skilled care use by their clients. Offsetting potentially negative implications for the TBAs reputation and future income may be an important part of programs that use TBAs to promote postnatal care attendance. TBAs expressed a desire for cordial relationships with and support from skilled health workers. This desire for recognition and support from formal health systems has also been reported by studies from Ghana, Honduras, and Somaliland [23–25]. Acceptance by formal health systems and creating an enabling health facility environment were factors that encouraged TBA referrals in a qualitative evaluation of a program in Somaliland [23]. In this study, health workers cited examples where integrating TBAs into service delivery or health care decision making appeared to encourage postnatal care referrals. Nigerian skilled health workers in other studies have also expressed support for recognition and integration of traditional birth attendants, while recognizing the need to curtail harmful practices [26]. Another suggestion for motivating referrals by TBAs was the use of financial and non-financial incentives. In Nigeria and Somaliland, qualitative evaluations indicate that monetary incentives may have increased the number of clients with complications who were referred to facilities postnatally [23, 27]. In a recent Kenyan field experiment, individual financial incentives were not effective and the intervention was modified to pay all TBAs in the village if any client from that village attended a facility, regardless of whether any TBA name was provided as a referrer [27]. TBAs in this study also expressed interest in non-financial incentives that would increase their capacity to provide quality services, such as maternal health care equipment and supplies.

Motivating TBAs to refer clients may be insufficient to increase maternal postnatal care utilization. In Guatemala, one quarter of the clients did not comply with the postnatal care referral by TBAs citing inadequate financial resources, lack of confidence in facility services, and difficulty with transport [21, 22]. In this study, TBA clients highlighted factors that informed their preference for the care TBAs provide over skilled care before, during, and after delivery. These factors may lead to non-compliance with postnatal care referrals by TBAs. There is near-consensus over the fact that TBAs provide respectful maternal care relative to skilled health providers [16, 17, 20, 28–30]. A synthesis of qualitative evidence from low and income countries reveals that disrespectful maternal care is a barrier to the use of facility-based delivery in many contexts [31]. Thus, the renewed attention accorded to the promotion of respectful care during pregnancy in recent years is potentially a significant step towards increasing skilled and facility-based maternal health care use [32]. Another attraction of TBA services noted in this study is the affordability relative to facility care and the possibility of paying after receiving antenatal and delivery services from TBAs (rather than prior to care as in facilities). Maternal clients considered cost of treatment and transport to be a barrier to receiving maternal postnatal care. In the most recent Nigerian Demographic and Health Survey, lack of financial resources for treatment payments was the highest reported barrier to the use of facility care (in 42% of respondents), followed by far distances from health facilities (28%) [3]. Studies of client preference for TBA services from Indonesia, Sierra Leone, and Zambia also report that affordability is one of the reasons why TBA service is preferred over skilled or facility maternal care, particularly in rural and remote areas [17, 20, 28, 29]. At the time of the interview, every client had taken her newborn to the facility for neonatal immunizations, despite the financial implications, yet only three received maternal postnatal care. Many primary health care centers in Nigeria are staffed by one or two health workers that hold antenatal, postnatal, and immunization clinics on different days of the week, so that mothers make multiple visits to receive care for themselves and their babies postnatally. Therefore, while maternal understanding of the importance of postnatal care for her wellbeing may be useful in increasing use, it has been recommended elsewhere that care for the mother and newborn also be integrated into one visit [2].

The strengths of this study include conducting focus group discussions in the local language, in addition to English, as several clients and TBAs communicate primarily in Igbo language and have no formal education. An open coding approach enabled the study team to focus on the perspectives of study participants and to avoid imposing preconceived categories during data analysis [11]. This study has several weaknesses. The focus groups, while in-depth, included a total of 28 participants, due to funding constraints. The study may have benefitted from increasing the number of focus groups with each type of study participant. The clients in this study were identified by TBAs and may have been purposively selected based on their positive disposition towards the TBAs services. This has implications for generalizability of the study findings to TBA clients more generally. This study may also have benefitted from participant observation of interactions between TBAs, TBA clients, and health workers, for comparison with FGD accounts. The study findings may also have limited application to other locations as TBA practices may vary. Future research on postnatal care attendance and referrals may also benefit from the perspective of health policy makers and other members of the household that often participate in maternal health care decision-making such as husbands.

## Conclusions

While TBAs may face potential negative social and economic consequences with postnatal care referrals, there are opportunities to engage TBAs in routine postnatal care referrals to facilities in Nigeria by using incentives and promoting a cordial relationship between TBAs and skilled health workers. These options may be accompanied by efforts to improve the service experience of mothers in facilities, including protecting the right to respectful maternal care and making services affordable. Further research is needed on the interactions between postnatal maternal complications, TBA referral behavior, and maternal perception of TBA competence.

## Additional file

Additional file 1: Summary of focus group discussion topic guides by participant type. Table summarizing focus group discussion topic guides by participant type (DOCX 17 kb)

## Abbreviations

COREQ: Consolidated criteria for reporting qualitative research
FGD: Focus group discussion
TBA: Traditional birth attendant
