# Supplementary material for: “Once the delivery is done, they have finished”: a qualitative study of perspectives on postnatal care referrals by traditional birth attendants in Ebonyi state, Nigeria
Source: BMC Pregnancy Childbirth. 2017 Dec 19;17:429. doi: 10.1186/s12884-017-1616-x (PMC5737984; doi:10.1186/s12884-017-1616-x)
Supplement: Additional file 1: — Summary of focus group discussion topic guides by participant type. Table summarizing focus group discussion topic guides by participant type (DOCX 17 kb) [file 12884_2017_1616_MOESM1_ESM.docx]

Summary of focus group discussion topic guides by participant type

| Participant Type | # | Topic Guide Themes |
| --- | --- | --- |
| Health Workers | 1 | Necessity for maternal and neonatal postnatal care |
|  | 2 | Content of postnatal care consultation |
|  | 3 | Scope of TBA practice in maternal health care |
|  | 4 | Appropriate role of TBA in maternal health care |
|  | 5 | Relationship between formal health workers and TBAs |
|  | 6 | Ways health workers can negatively or positively influence TBA roles |
|  |  |  |
| TBA Delivery Clients | 1 | Maternal and neonatal postnatal care - perceived rationale, location, timing, provider |
|  | 2 | Perception of TBA in community - roles, relative skill compared to health workers, credibility |
|  | 3 | Referrals by TBA for antenatal, delivery, and postnatal care - effect on decisions, perception of TBA |
|  | 4 | Payment to TBAs for referrals - effect on decisions, perception of TBA |
|  | 5 | Factors that negatively or positively influence maternal postnatal care attendance |
|  |  |  |
| TBAs | 1 | Scope of TBA practice in maternal health care |
|  | 2 | Content and timing of maternal health care practice |
|  | 3 | Motivation for maternal health care practice |
|  | 4 | Perception of care quality relative to formal health workers |
|  | 5 | Current practice of and motivations for referrals - antenatal, delivery, and postnatal care |
|  | 6 | Relationship between formal health workers and TBAs |
|  | 7 | Perception of potential role as advocate for skilled postnatal care |
|  | 8 | Factors that might negatively or positively influence role as advocate for skilled postnatal care |
|  |  |  |
|  | 9 | Payment to TBAs for referrals - potential effect on referrals |
|  | 10 | Willingness to share information on referrals and deliveries - potential effect of payment |
